# Polycystic ovarian syndrome: Environmental/occupational, lifestyle factors; an overview

**DOI:** 10.4274/jtgga.galenos.2019.2018.0142

**Published:** 2019-11-28

**Authors:** Chaoba Kshetrimayum, Anupama Sharma, Vineet Vashistha Mishra, Sunil Kumar

**Affiliations:** 1 Department of Reproductive and Cytotoxicology, ICMR-National Institute of Occupational Health, Ahmedabad, India; 2 Department of Gynecology, Institute of Kidney Diseases, Ahmedabad, India; 3 Former, Scientist G & Director-in-Charge NIOH (ICMR), Ahmedabad, India; # PhD Scholar, Life Science, Gujarat University, Ahmedabad, India

**Keywords:** Polycystic ovary syndrome, environmental, occupational, hyperandrogenism, anovulation, genetic factors, androgenization, infertility

## Abstract

Polycystic ovary syndrome (PCOS) is a multifaceted disease of women with infertility that has diverse etiologic factors. Some women may have only a few PCOS-linked symptoms or mild symptoms, whereas others will have severe or all PCOS-linked symptoms. Therefore, PCOS symptoms can differ among women. PCOS is a state of hormonal imbalance, excess terminal hair (hirsutism), hair loss (alopecia), menstruation impairments, metabolic disorders, and cystic appearance on the ovaries. The cysts hamper ovulation, thus reducing the ability of women to become pregnant and result in infertility. The available data suggest that PCOS might originate in utero and the phenotypic appearance of PCOS symptoms may be developed in later life, which could be linked with host factors (endogenous) and exogenous factors like lifestyle, and dietary, environmental or occupational factors. Based upon the available information, it can be postulated that prenatal exposure to excessive androgens might be responsible for androgenization of the fetus, which in turn may alter the program of differentiating target tissues and the phenotypic characteristics of PCOS can be persuaded by exposure of female offspring to various endogenous and exogenous factors at later life. Genetic/host and environmental/lifestyle factors might be related to the pathophysiology of PCOS after prenatal exposure to androgen. Additional studies are necessary to understand the exact mechanism responsible for the manifestation of PCOS because it is a very important issue in female reproduction.

## Introduction

Polycystic ovary syndrome (PCOS) is a problem of teenage girls or women in which their hormonal levels are impaired. It can cause problems in the menstruation cycle and difficulty in conceiving. In this disease, many small cysts develop in the ovaries of women, hence its name. PCOS is globally considered to be the foremost reason for infertility in women. PCOS is a multifaceted disease with various etiologic factors, which might be related with the disease or exaggerate the problem or induces PCOS phenotypic characteristics in women in adulthood. Owing to anovulation, it is the main cause of infertility and most common endocrine disorder of women. It affects the lives of women from *in utero* life to till death.

PCOS is also linked with several health hazards, which in turn elevate morbidity, impair quality of life, and increase mortality rate (1). The prevalence of PCOS differs because several diverse criteria are used for the diagnosis of PCOS by different investigators and diverse norms for diagnosis are also suggested by various organizations. It is an endocrine syndrome with menstruation irregularities, hyperandrogenism, and polycystic ovaries of women (2). Based upon the criteria of diagnosis for PCOS by the European Society for Human Reproduction & Embryology/American Society for Reproductive Medicine, the prevalence of PCOS is about 15-20% (2). Later, a meta-analysis was conducted by including studies that were published from 2006 to 2011 from Iran. The prevalence rate based on the criteria of the National Institute of Child Health and Human Disease of the United States was 6.8%, 19.5% based on Rotterdam norms, and 4.4% based on ultrasound criteria (3). About 5 to 10% of reproductive-aged women of Indian sub-continent are reported to be affected with PCOS (4).

Polycystic ovaries, chronic anovulation, and hyperandrogenism are the distinctive characteristics of PCOS and with the existence of insulin resistance, hyperinsulinemia, hypertension, abdominal obesity, and dyslipidemia are responsible for long-term serious outcomes such as endometrial hyperplasia, type 2 diabetes mellitus, and coronary artery disease (4). They also mentioned that it is only the interaction of environmental factors (obesity) with genetic factors that result in the appearance of PCOS, even though a woman may be genetically susceptible for the development of PCOS (4).

Many women may have PCOS illnesses and live with it without being diagnosed clinically. March et al. (5) drew attention to this important issue of PCOS diagnosis in the community and mentioned that about 68% of women with PCOS had not been tested for PCOS in the past. This showed the seriousness of the diagnostic problem of PCOS in the community. Further, the appearance of PCOS may also affect the mental health of the subjects, which might also be related to the psychiatric problems among such patients due to the difficulty in conceiving. The prevalence of depression and anxiety was 27.5% and 13.3%, respectively, in subjects with PCOS as compared with 3.0% and 2.0% among control subjects (6). Further, about 65-70% of women with PCOS had compensatory hyperinsulinemia and insulin resistance (7). In addition, an inherent ovarian defect (possibly genetically) existed in PCOS women, which makes the ovary vulnerable to insulin stimulation of androgen production. However, limited data/evidence also suggests hyperinsulinemia might stimulate androgen production in ovaries (8). Later, Baptiste et al. (9) described several steps for the appearance of PCOS: (a) enzymatic default in the ovarian and/or adrenal steroidogenesis; (b) variation in the gonadotropin-releasing hormone that encourages luteal hormone secretion; or (c) amendments in insulin actions leading to insulin resistance with compensatory hyperinsulinemia. Some women with the characteristic of PCOS do not show insulin resistance, this advocates the hypothesis of a genetic predisposition to PCOS. This would be exhibited by the progression of insulin resistance and compensatory hyperinsulinemia in the majority of women with PCOS, but not all women with PCOS (9).

In addition to the endogenous or host factors, it is also recognized that some factors such as chemical, physical, dietary, lifestyle, occupational, and environmental factors are accountable for hostile consequences on human male and female reproduction and they might also affect pregnancy and its outcome. Several reports and reviews on the role of occupational and environmental aspects on different aspects of human reproduction (10,11,12,13,14) and controlled experimental studies on certain environmental and lifestyle factors on reproductive health have been published (15,16,17) from this laboratory.

The hazard factors of PCOS comprised menstrual cycle impairment [odds ratio (OR)=5.8], bad mood (OR=2.8), family history of diabetes (OR=7.0), infertility in the family (OR=11.9), mother menstrual irregularity (OR=2.5) and physical exercise deficiency (OR=1.8) (18). The existing data on various environmental factors suggest their potential contribution in the etiology, prevalence, and modulation of the syndrome. There is evidence that advocates that environmental factors might play a significant role in deteriorating reproductive health and some environmental factors are vital behind the deterioration, including environmental toxins, diet and nutrition, socioeconomic status, and geography (19). Nevertheless, research/data on these environmental factors with reference to the causation of PCOS are limited or inconsistent and further well-planned studies are needed.

This overview is furnished based on available information on the role of occupational, environmental, and lifestyle factors in PCOS. The information was collected through searching various websites such as Google, PubMed, Medline, Toxline, and other websites and consulting related books. This overview is separated into various segments and the first section deals with the existing information on the recognized host/genetic factors associated with PCOS. The second and third sections deal with occupational/chemical exposures and lifestyle factors that might be associated with PCOS. In addition, some light is also shed on oxidative stress in the occurrence of PCOS. The majority of on-hand reviews on PCOS are available on the host/genetic factors rather than the role of both occupational/environmental toxicants exposure and lifestyle influences and PCOS.

**Figure 1:**
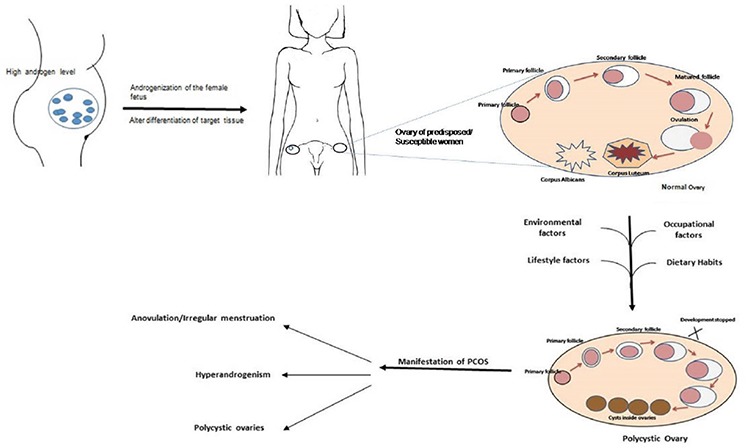
Possible factors of occurrence of PCOS PCOS: Polycystic ovary syndrome

In this review, importance has been given to parental environmental exposure and lifestyles factors in PCOS, covering mainly human studies. The possible etiologic factors related to the progression/development of PCOS are depicted in Figure 1.

## Results and Discussion

The exact mechanism for the manifestation of PCOS is not yet completely understood. The syndrome appears to involve genetic, environmental, dietary, metabolic components. The origin of PCOS starts from early life in the mother’s womb, extending throughout the lifecycle, and environmental insults and lifestyle issues may affect vulnerable women, leading to the occurrence of phenotypic characteristics of PCOS. Diet seems to be one of the foremost environmental determinants for the occurrence of PCOS. Hormone levels are imbalanced among women with PCOS. Generally, women with PCOS have elevated male hormone (androgens) and lower levels of the female hormone (estrogen). High androgen concentrations can also have a significant impact on female reproductive development and function.

PCOS seems to be one of the ancient ailments that continued through human evolution (20). A report indicated that the anti-mullerian hormone (AMH) level was 2-3 three times higher in women with PCOS as compared with normal levels (10±2.2 ng/mL), and this high AMH level is a good indicator of infertility and PCOS (21). Further, women with PCOS are stated to have significantly higher levels of serum AMH as compared with controls, and the occurrence of negative and positive correlations with other hormonal parameters showed involvement of AMH, at least in part, in the manifestation and development of PCOS (22). The data available on serum levels of AMH in subjects with PCOS suggest its use as a diagnostic biomarker and it can serve as a reliable tool to describing the severity of the disorder, monitoring, and forecasting a prognosis of the diseases.

Recently it has been stated that AMH is raised and strongly associated with several reproductive, metabolic, and endocrine impairments in subjects with PCOS. AMH also has an inhibitory function in follicular growth and recruitment. The follicle-stimulating hormone (FSH)-induced aromatase production due to the preventive action of AMH probably contributes to hyperandrogenism, which further increases the insulin resistance in women with PCOS. In addition, elevation in serum AMH levels is extrapolative of poor treatment response in women with PCOS i.e. loss of weight, induction of ovulation, and laparoscopic ovarian drilling, whereas improvement in several other clinical parameters after treatment was related to declining serum AMH levels. This advocates a significant role of this hormone in the pathophysiology of PCOS (23).

The pregnancy-related difficulties were reported to be more frequent in women with PCOS. Various etiologic factors involved in PCOS and allied co-morbidities may also be connected to compromised pregnancy and/or its outcomes. A possible relationship between genetic, environmental, clinical and biochemical, and dietary factors is involved in the occurrence of this complex syndrome, with pregnancy complications and its outcome (24). In this overview, more emphasis has been given on lifestyle, occupational, and environmental issues in PCOS. However, some information on other factors is also incorporated to understand the overall possible causative factors/mechanism connected with PCOS.

### Endogenous/host/genetic factors

Several genetic/host factors might be related to the development/occurrence of PCOS. The genetics behind the appearance of PCOS are yet not fully understood, but ample evidence has been provided of their role in PCOS. Further, a considerably higher number of women in the PCOS group was reported to have a family history of diabetes and some women in this group also had a self-history of diabetes, whereas no women were diabetics in the controls (25). The presence of type 2 diabetes is noticeably higher in middle-aged women with PCOS, which suggests that body mass index, glucose, and sex hormone-binding globulin levels are connected to the risk stratification of PCOS (26).

Based upon animal studies and reinforced by clinical studies, it is reported that PCOS has its origin in fetal life, and exposure to excess androgens from the time of the growth of the ovaries in fetal life to the commencement of puberty leads to distinctive features of PCOS, along with irregularities in luteinizing hormone secretion and insulin resistance (27). PCOS arises from ancestral gene variants, which are an ancient syndrome. Such ancient genes were likely to be transmitted trans-generationally through offspring conceived amongst fertile carrier males and sub-fertile affected females (20). PCOS is a heterogeneous syndrome and commonly determined by the implication of two vital factors i.e. hyperandrogenism and insulin resistance (28). Alterations in genes that control the ovarian steroidogenesis are possibly the foremost contributing factors of hyperandrogenism. Insulin resistance may be due to different gene variations such as insulin receptor substrate-1 and 2, calpain-10, and peroxisome proliferator-activated receptor (28).

In most subjects, PCOS seems to be determined by the association of gene polymorphisms common in the general population, but gene polymorphisms alone are incapable of determining phenotypic consequences. The heterogeneity of the ailment can be explained by numerous combinations of multiple gene polymorphisms and environmental factors (28). Based upon numerous available studies, a sturdy genetic element is evident for the etiology of PCOS. Keeping in view the vast genetic and phenotypic heterogeneity of PCOS and inadequate large cohort studies to identify precise causative genes, only a few definite outcomes have been provided that concluded the heterogeneity of the disease and inadequate sample sizes complicated the identification of exact genes responsible for PCOS (29). Heritable predispositions have been stated in the occurrence of PCOS, and a genome-wide association study (GWAS) regarding PCOS showed evidence for the genetic mechanisms in the pathophysiology of PCOS. They suggested that studies using innovative techniques such as next-generation sequencing would be beneficial to understand more about underlying variants for PCOS (30). Further, PCOS is reported to be linked with oligomenorrhea, hirsutism, hyperandrogenism, insulin resistance, obesity, and risk of type 2 diabetes mellitus by ~ 7-folds (31).

Most women with PCOS (both obese and lean) have insulin resistance. The mini-satellites of the insulin gene, specifically class III alleles and III/III genotypes, are connected to the risk of type 2 diabetes and determine the predisposition to anovulatory PCOS. In addition, the appearance of estrogen receptor and 5-alpha-reductase gene (*SRD5A1-2* genes) activity in granulosa and theca cells indicated a significant variation in the expression of estrogen receptor (ER) alpha and ER beta in PCOS that may be linked with anomalous follicular development (31). The higher frequency of individuals with PCOS and the extensive range of phenotypic appearances of the disease can be elucidated by the interaction of several main genes with environmental factors (32). However, some confirmation of familial segregation and clustering of the ailment in the first-degree relatives of women with PCOS has been demonstrated with no pattern of inheritance. The existing genetic studies put forward a strong familial element and PCOS is considered a polygenic trait, which might be a consequence of the interaction of vulnerable and defensive genomic variants and environmental aspects during pre or postnatal life (32). Based upon all these studies, it can be inferred that PCOS has a sturdy genetic element in the occurrence of this syndrome along with other factors.

Duniaf and Thomas (33) mentioned that family history showed a genetic vulnerability of PCOS. Women with PCOS with insulin resistance have an ~ 50% chance of their sister having polycystic ovaries; hyperandrogenemia and high low-density lipoprotein are consistent with genetic traits. Family-based studies on linkage and association of factors have implicated numerous genes in the causation of PCOS (33). Further, it is apparent that genome-wide association studies have evolved powerful means for studying the genetic architecture of human disease (34). PCOS reduces fertility without changing in prevalence and it was considered as an evolutionary paradox (35). Overall, 17 single nucleotide polymorphisms (SNPs) were identified by GWAS studies, which were related to PCOS, with different allele frequencies, ethnicity-related, in 11 susceptible loci. The authors further examined phenotype-genotype correlations of PCOS *in silico* and suggested that PCOS was a genetic gradient that resulted from genetic drift due to the consequences of a series of events that took place in early human migrations (35). A few GWAS studies were published on PCOS from diverse areas and different ethnic groups of the world e.g. European countries, China, Korea. The GWAS study on PCOS was conducted among Han Chinese and acknowledged robust evidence of an association between PCOS and three loci: 2p16.3; 2p21 and 9q33.3. These results offer a new understanding of the pathogenesis of PCOS (36).

Lee et al. (37) recognized a novel locus with genome-wide implication and seven moderately linked loci in Korean women with PCOS. The strongest relationship was found on chromosome 8q24.2, and other association signals were situated at 4q35.2, 16p13.3, 4p12, 3q26.33, 9q21.32, 11p13, and 1p22. The strongest signal was situated upstream of KHDRBS3, which is linked with telomerase activity, and that might be resulted to PCOS and associated phenotypes (37). Later, common genetically susceptible loci were also reported in European lineage women and three loci were reported to have genome-wide implication in a case-control meta-analysis i.e. two novel loci mapping chr 8p23.1 and chr 11p14.1, and also the chr 9q22.32 locus found earlier in Chinese women with PCOS. PCOS diagnosis and LH levels were strongly linked with the chr 11p14.1 SNP, rs11031006, in the region of the *FSH B polypeptide* gene (38). The genetic risk calculated by GWAS studies was significantly connected with PCOS and associated clinical features (39). There is a report that indicated that androgen metabolism was deteriorated in women with PCOS, thus the CYP19 gene, which is associated in this pathway, can be a novel gene for investigation (40). Studies also revealed an association between an SNP of the *CYP19* gene in hyperandrogenism and PCOS in some ethnic groups. The studies further stated that among Iranian women, variants of SNP rs.2414096 in CYP19 could be responsible for the occurrence of PCOS (40). All these studies clearly suggest the role of genetic factors in the appearance of PCOS.

### Environmental/Occupational factors

Exposure to some of lifestyle, occupational, environmental factors may enhance the elevation in the occurrence of PCOS or exaggerate the incidence of PCOS and/or phenotypic signs of PCOS, but the cause-effect relationship of these aspects with PCOS is still lacking or inadequate. There are also inadequate or inconsistent studies on exposure to environmental/occupational/lifestyles factors with regards to PCOS. Further, women are exposed to several chemicals during their day-to-day activities without their knowledge and some of these chemicals may have estrogenic or anti-estrogenic, androgenic or anti-androgenic properties. These chemicals act at a very low dose and are known as endocrine disruptors (EDs). EDs might act through membrane-bound estrogen-receptors; estrogen-related receptors; nuclear receptors; interaction with targets in the cytosol and variations in endogenous hormones metabolism; cross-talk between genomic and non-genomic pathways, interfering with feedback regulation; cross-talk with estrogen receptors after binding on other receptors and alterations in neuroendocrine cells; and DNA methylation or histone alterations (41). There is a report that indicated that experimental exposure to industrial endocrine disruptive chemicals contributed to the worsening of normal reproductive function and metabolic regulation, perhaps contributing to the growth of or enhancing PCOS-resembling clinical ailments. Industrial chemicals may also contribute to the causative role of a hostile environment to unveil PCOS characteristics in genetically susceptible individuals or further deteriorate the hormonal steadiness and fertility status of women with PCOS (42).

In addition, hormonal activity is affected due to exposure to chemicals in the womb, which may exaggerate the development of PCOS. The elevated concentrations of bisphenol-A (BPA) in women with PCOS and a noteworthy positive relationship between androgens and BPA suggests a probable role of this endocrine disruptor in the causation of PCOS (43). Further, elevated serum BPA levels were found in teenage girls with PCOS, independent of obesity, more than in the controls. BPA levels were also evidently associated with androgen concentration, leading to the inference that BPA might have a considerable role in the occurrence of PCOS in teenage girls (44). Exposure to EDs that mimic natural hormones during prenatal development might contribute to deviating the fetal programming of target tissues, which may be associated with PCOS and may have several potentially adverse trans-generational health effects (45). Chronic or acute exposure to advanced glycation end-products and EDs during various stages of the life cycle may result in the interruption of hormonal homeostasis, which is linked to the deterioration of reproductive functions. They may also interfere with metabolic changes such as insulin resistance, obesity, and compensatory hyperinsulinemia, which can contribute to PCOS consequences such as cardiovascular disease and type 2 diabetes (45). However, phthalic acid esters, BPA and octylphenol, do not induce an apparent effect on the manifestation of PCOS or contribute to insulin resistance, but octylphenol may play a considerable role in insulin resistance in subjects with PCOS (46).

Vagi et al. (47) also conveyed that subjects with PCOS might have different environmental contaminant profiles from controls, and reported that women with PCOS had higher serum concentrations of perfluoro octanoate and perfluoro octane sulfonate, and lower concentrations of mono-n-butyl phthalate and mono-benzyl phthalate in urine. They also mentioned that more studies are required to confirm these findings. Environmental factors are likely to be related with the occurrence of PCOS. Women with PCOS were found to be consuming plastic-packaged food, eating fruit with pericarp, pesticide exposure, living close to a garbage heap, working at an acid plant, taking Chinese medicines, smoking, and ingesting alcohol more commonly than controls. Eating plastic-packaged food, eating fruit with pericarp, and alcohol consumption were independent hazards for the manifestation of PCOS (48). The authors further reported that relationships of these factors with PCOS should be confirmed by conducting additional studies.

Earlier it was also reported that environmental issues linked to PCOS were occupation, education, disposable plastic cups for drinking, indoor decoration, and cooking oil fumes, and all were significantly connected to PCOS (49). It is recognized that PCOS has the characteristic of endocrine disturbances, thus EDCs might be one of the underlying causes of PCOS. Based upon experimental studies, it was stated that BPA exposure in the perinatal stage, often at doses comparable to human exposure, interrupted ovarian and reproductive function. BPA seems to have obesogenic qualities, affecting standard metabolic function and the body becomes prone to being overweight. Cross-sectional studies suggested that PCOS women had higher BPA levels with respect to women with good reproductive health. They also suggested that additional investigations are required to extrapolate the mechanisms, wherein EDs might be linked with PCOS and critical time periods of EDCs exposure, which might have a trans-generational effect (50).

Recently, the relationship between PCOS and the anogenital distance (AGD) was explored by different investigators, which is a biomarker of androgen exposure in fetal development, and observed that subjects with PCOS exhibited higher AGD as compared with controls. This suggests that PCOS has an intrauterine origin, and fetal hormonal environment may be responsible for the advancement of PCOS in later life (51). It is acknowledged through animal studies that, prenatal exposure to high testosterone induces PCOS-like phenotypes, even though the etiology of PCOS is unfamiliar. Further, infant girls born to women with PCOS have longer AGD, which implies excess prenatal testosterone exposure with respect to girls born to women without PCOS (52). The prevailing information on prenatal exposure to three important categories of EDCs i.e. phthalates, BPA, androgenic EDCs, and the occurrence of PCOS and/or PCOS-linked anomalies were described thus: (1) maternal BPA exposure modifies sexual maturation and postnatal development in rodents; (2) gestational exposure to dibutyl phthalate and di-(2-ethylhexyl) phthalate induces polycystic ovaries and PCOS like hormonal profile; and 3) androgenic EDCs such as 3,4,4’-trichlorocarbanilide and nicotine, generate fetal hyperandrogenic environment. EDC exposure during prenatal growth may be accountable for altering fetal programming (53).

### Lifestyles and dietary factors

Lifestyles and dietary factors may indirectly contribute to the occurrence of PCOS because exposure to these factors has been linked with the appearance of PCOS in girls who are susceptible to PCOS. PCOS is a common ailment, and women with PCOS have reproductive, metabolic, and psychological consequences. Weight gain and obesity deteriorate the characteristic features of PCOS, while weight decline, diminishes the characteristic features of PCOS (54). The excess weight loss through lifestyle alteration leads to menstrual regulation and regulates reproductive outcomes in women with PCOS. The available data support that a moderate diet with carbohydrates, poly and mono-unsaturated fats, and a high content of fiber with lean protein sources, are beneficial to overall health parameters in women with PCOS. Further, the incorporation of exercise in daily life showed a positive effect on the clinical representations of PCOS (55). Therefore, management of PCOS must include better lifestyle approaches with regard to proper diet, exercise, optimization of body weight, and improving insulin sensitivity, to target PCOS-related health apprehensions.

Recently, lifestyle (diet and exercise) intervention was shown to recover levels of FSH, sex hormone binding globulin, androstenedione, total testosterone, free androgen index, and Ferriman-Gallwey scores in women with PCOS (56). Losing body weight and exercise are important factors reported to improve the condition of menstrual impairment, and infertility was noticed in obese women with PCOS (57). The lifestyle variation program with importance on behavioral management, dietary, and workout interventions has been described as being successful in lowering the risk of diabetes and metabolic syndrome in the general population and accomplishing an improvement in fertility outcomes in patients with PCOS (58). These data clearly exhibited the positive role of adopting a healthy lifestyle for managing PCOS to some extent.

### Trace and heavy metals in women with PCOS

Some metals in trace quantities are essential for various physiologic functions in the human body. Hence these are called essential trace metals. Essential trace and heavy metals were studied in human subjects with PCOS and serum copper (Cu), and zinc (Zn) concentrations were found to be significantly higher, whereas manganese (Mn) and lead (Pb) values were lower in subjects with PCOS. These findings should be explored further to find new insights into metals and PCOS (59). Further, no considerable differences in the median levels of barium, lead, cadmium, chromium, arsenic, strontium, gallium, and vanadium were reported amongst subjects with PCOS and controls. In contrast, serum nickel and copper concentrations were significantly higher, and Zn was significantly lower in subjects with PCOS compared with controls. Thus, metals such as copper and nickel might be implicated in the causation of PCOS and linked with impairment of reproductive hormone levels (60).

The relationship between hormonal impairments and the alterations of trace element (manganese), macro elements (magnesium and calcium), heavy metals such as cadmium, and lead in both obese and non-obese subjects with PCOS was studied and significantly higher blood Pb and Cd levels were found in subjects with PCOS (non-obese and obese) as compared with control subjects, and significantly low levels of magnesium, calcium, and Mn were recorded in subjects with PCOS (61). The serum FSH level was significantly lower in obese subjects with PCOS compared with control (obese and non-obese) subjects. A positive association was observed among serum testosterone and Cd levels in obese women with PCOS. This study established that elevated blood Pb and Cd concentrations and lower levels of serum calcium, magnesium, and Mn were observed in PCOS subjects as compared with controls (61). Later, Taher and Mhaibes (62) reported that serum copper and nickel levels were considerably elevated in subjects with PCOS, whereas the concentration of serum zinc was declined in subjects with PCOS (obese and non-obese) in comparison with controls (obese and non-obese). Further, Sedighi et al. (63) compared the lifestyle of women with PCOS and reported a noteworthy association between the manifestation of PCOS and improper diet, low physical activity, but no association with unhealthy behaviors. The data on lifestyle, diet, and some metals suggest that these factors may also have some role in the occurrence of PCOS phenotypic symptoms and weight management, healthy lifestyle, and regular exercise might be beneficial in the reduction of PCOS linked features in young adult girls.

### Oxidative stress and PCOS

In addition to androgenization of female fetus, genetics, host, dietary and other environmental, lifestyle factors, oxidative stress might also be related in the occurrence of PCOS. Oxidative stress is a phenomenon of disproportion between the excessive generation of free radicals and the balancing system of antioxidant status in the body to detoxify these extra free radicals efficiently. Based upon a review on oxidative stress indicators in women with PCOS, it was mentioned that circulating indicators of OS were unbalanced in women with PCOS independent of excess weight, suggesting that OS might play a significant role in the occurrence of PCOS (64). An increased level of ROS and myeloperoxidase in subjects with insulin resistance and PCOS were also reported. Further, inflammation in subjects with PCOS brings about leukocyte-endothelium interactions and a concurrent elevation of tumor necrosis factor-α, interleukin-6, leukocytes, and adhesion molecules i.e. E-selectin, ICAM-1, and VCAM-1, and these situations are heightened by the existence of insulin resistance (65). Further, OS is a significant factor of the cardio-metabolic risk found in women with PCOS and adjusting oxidative stress with supplementation of antioxidants along with measuring antioxidant status could have a valuable effect on OS-induced hyperandrogenism and insulin resistance found in non-obese women with PCOS (66).

Earlier, González et al. (67) reported that ROS production in response to hyperglycemia from mononuclear cells was elevated in subjects with PCOS, which are independent of obesity. The resultant OS might subscribe to a proinflammatory state, which encourages hyperandrogenism and insulin resistance in women with PCOS. In addition to hormonal imbalances, defects in insulin signaling and dysfunction of adipose tissue, oxidative stress has been robustly implicated in the etiology of occurrence of the PCOS. Oxidative stress, with other etiologic factors of PCOS and involvement of environmental factors, leads to a hostile redox status that stigmatizes the normal progression of PCOS (68). The strong association of insulin resistance with OS at the visceral adipose tissue level was also observed indicating local OS and defects of insulin signaling in adipose tissue may play a vital role in the causation of PCOS (69) along with other genetic, host, and dietary or environmental factors.

The program of differentiating target tissues to the occurrence of PCOS phenotypic characteristic later in life might result from the prenatal androgenization of the female fetus due to both genetic and environmental factors and their interaction (70). The data available from both experimental and clinical studies suggest that maternal hyperandrogenism is a causative factor of PCOS, and variations in the gestational endocrine environment due to hyperandrogenism in women with PCOS in pregnancy, may play a vital part in the vertical transmission of PCOS. The scarcity of data in humans at early gestational stages has been emphasized, and the importance of experimental data to understand the cellular and molecular mechanisms involved in the programming of adult diseases. The two-hit hypothesis was proposed for the appearance of PCOS i.e. perinatal organizational and postnatal activation events (71).

Based upon available clinical and experimental data, one can infer that PCOS is a consequence of androgenization as well as alteration of the programming of target tissue differentiation during fetal development, metabolic disorders, exposure to EDs during pre and postnatal development, along with lifestyle and dietary factors in later life are associated with development of PCOS phenotypic symptoms; this also supports the two-hit hypothesis i.e. prenatal organizational and post-natal activation as reported earlier (71). The management of PCOS can be achieved through better lifestyles such as appropriate diet, exercise, optimization of body weight, and improving insulin sensitivity to control this syndrome.
